# Role of Quercetin in Modulating Chloride Transport in the Intestine

**DOI:** 10.3389/fphys.2016.00549

**Published:** 2016-11-23

**Authors:** Bo Yu, Yu Jiang, Lingling Jin, Tonghui Ma, Hong Yang

**Affiliations:** ^1^Liaoning Provincial Key Laboratory of Biotechnology and Drug Discovery, School of Life Sciences, Liaoning Normal UniversityDalian, China; ^2^College of Basic Medical Sciences, Dalian Medical UniversityDalian, China

**Keywords:** CaCC, ANO1, modulator, quercetin, short-circuit current

## Abstract

Epithelial chloride channels provide the pathways for fluid secretion in the intestine. Cystic fibrosis transmembrane conductance regulator (CFTR) and calcium-activated chloride channels (CaCCs) are the main chloride channels in the luminal membrane of enterocytes. These transmembrane proteins play important roles in many physiological processes. In this study, we have identified a flavonoid quercetin as a modulator of CaCC chloride channel activity. Fluorescence quenching assay showed that quercetin activated Cl^−^ transport in a dose-dependent manner, with EC_50_ ~37 μM. Short-circuit current analysis confirmed that quercetin activated CaCC-mediated Cl^−^ currents in HT-29 cells that can be abolished by CaCC_inh_-A01. *Ex vivo* studies indicated that application of quercetin to mouse ileum and colon on serosal side resulted in activation of CFTR and CaCC-mediated Cl^−^ currents. Notably, we found that quercetin exhibited inhibitory effect against ANO1 chloride channel activity in ANO1-expressing FRT cells and decreased mouse intestinal motility. Quercetin-stimulated short-circuit currents in mouse ileum was multi-component, which included elevation of Ca^2+^ concentration through L-type calcium channel and activation of basolateral NKCC, Na^+^/K^+^-ATPase, and K^+^ channels. *In vivo* studies further revealed that quercetin promoted fluid secretion in mouse ileum. The modulatory effect of quercetin on CaCC chloirde channels may therefore represent a potential therapeutic strategy for treating CaCC-related diseases like constipation, secretory diarrhea and hypertension. The inverse effects of quercetin on CaCCs provided evidence that ANO1 and intestinal epithelial CaCCs are different calcium-activated chloride channels.

## Introduction

Epithelial tissues regulate ion intake and secretion through ion channels and transporters. Epithelial chloride secretion provides the main driving force for water secretion. The secretion of chloride ion occurs via activation of chloride channels in the apical membrane of intestinal epithelial cells. Cystic fibrosis transmembrane conductance regulator (CFTR) and calcium-activated chloride channels (CaCCs) are the main chloride channels in the luminal membrane of enterocytes (Riordan et al., 1989; Hartzell et al., 2005). CFTR is a cAMP-dependent chloride channel expressed in crypt cells, and is a member of the ATP-binding cassette (ABC) membrane transporter gene superfamily. Calcium-activated chloride channels exist in various excitable and non-excitable cells, and they play an important role in many physiological processes, such as protein secretion and the vectorial transport of salt (Kunzelmann et al., 2007), prevention of multiple fertilization (Runft et al., 1999), smooth muscle contraction (Angermann et al., 2006), olfactory signal transduction (Matthews and Reisert, 2003), neurons excitation and repolarization of cardiac action potential (André et al., 2003; Guo et al., 2008), but the molecular identity of intestinal epithelial CaCCs remains unclear.

The first calcium-activated chloride channel identified was ANO1 (also known as TMEM16A) (Caputo et al., 2008; Schroeder et al., 2008; Yang et al., 2008). ANO1 is expressed abundantly in the intestinal pacemaker Cajal cells and participates in the contraction of smooth muscle in the intestine (Huang et al., 2009; Hwang et al., 2009; Ferrera et al., 2010). At present, the physiological effect of ANO1 on the secretion of liquid in intestinal epithelium is still controversial. Kunzelmann et al. proposed that ANO1 is the major calcium-activated chloride ion channel in intestinal epithelium, because ANO1 is expressed in mouse colonic epithelia (Ousingsawat et al., 2009) and the loss of ANO1 causes a defect in epithelial Ca^2+^-dependent chloride transport. Nevertheless, using ANO1-specific inhibitor, Verkman and coworkers found that ANO1 contributes only a minor component in Ca^2+^-dependent Cl^−^ current in enterocytes, therefore suggesting that different types of CaCCs may exist in enterocytes (Namkung et al., 2011a). Gastrointestinal epithelial Ca^2+^-activated chloride channel (CaCC_GI_) is considered to be pre-dominantly involved in Ca^2+^-activated chloride secretion, but the molecular identity of CaCC_GI_ still remains unknown.

CaCC modulators are important for illustrating the gating mechanism of CaCC, and selective CaCC modulators provide effective probes for studying the physiological and pathophysiological functions of CaCC chloride channels. Moreover, highly active CaCC modulators also provide new therapeutic strategy for the treatment of CaCC-related diseases. Thus, it is vital to discover new CaCC small molecule modulators. In 2008, using a phenotye-based fluorescence screening model, Verkman and coworkers identified the aminothiophene compound CaCC_inh_-A01 as a CaCC inhibitor from a combinational small molecule library (De La Fuente et al., 2008). The same group subsequently also identified several CaCC inhibitors with different structures from natural products, including tannic acid, digallic acid, gallotannin, EGCG and ECG (Namkung et al., 2010). In 2011, an ANO1-specific inhibitor called T16A_inh_-A01 was also identified using similar cell-based fluorescence screening model, along with ANO1 activator E_act_ and potentiator F_act_ (Namkung et al., 2011b). However, fewer CaCC activators have been identified compared to inhibitors.

In previous studies, we have set up a high-throughput screening-based natural compound identification strategy (Zhang et al., 2014; Chen et al., 2015). Based on this strategy, we have found a number of natural compounds with CaCC activation activity including quercetin. The aim of the present study was to systematically investigate the modulation effect of quercetin on intestinal CaCC chloride channels.

## Materials and methods

### Cell lines, animals, and compound

HT-29 cells transfected with or without the halide sensor YFP-H148Q/I152L fluorescence protein were cultured in RPMI 1640 medium (Sigma Chemical Co, St. Louis, MO. U.S.A.) supplemented with 8% fetal bovine serum, 2 mM L-glutamine, 100 U/ml penicillin, and 100 μg/ml streptomycin in a 37°C incubator with 5% CO_2_ and 95% humidity.

Fisher rat thyroid (FRT) cells stably transfected with ANO1 were cultured in Coon's modified F12 medium (Sigma Chemical Co, St. Louis, MO. U.S.A.) supplemented with 10% fetal bovine serum, 2 mM L-glutamine, 100 U/ml penicillin, and 100 μg/ml streptomycin in a 37°C incubator with 5% CO_2_ and 95% humidity.

Male ICR mice (8–10 weeks) were fed with a chow diet and kept under specific pathogen-free conditions at Dalian Medical University (Permit Number: SCXK liao 2008-0002, 2013-0003).

Indomethacin and amiloride were all purchased from Sigma (Sigma Chemical Co, St. Louis, MO. U.S.A.). ATP and NaI were purchased from Sangon (Sangon Biotech Co., Ltd, Shanghai). Carbachol (CCh) was obtained from EDM chemicals, Inc. (San Diego, CA). E_act_ and CaCC_inh_-A01 were purchased from Chembest (Chembest research laboratories Co., Ltd, Shanghai). Amphotericin B was bought from Solarbio (Solarbio Science and Technology Co., Ltd.). Quercetin was obtained from Shanghai Tauto Biotech Co., LTD (Shanghai, China). CFTR_inh_-172 was synthesized as described by Garcia et al. (2009). T16A_inh_-A01 was provided by Dr. A.S.Verkman (University of California, San Francisco, USA).

### Iodide influx measurement

Fluorescence assays were performed using a FLUOstar Optima fluorescence plate reader (BMG Labtechnologies, Durham, NC) equipped with syringe pumps and fixed excitation/emission (500 ± 10 nm/535 ± 15 nm) filters (Chroma, Brattleboro, Vernont, USA). YFP-expressing HT-29 cells were plated in 96-well black-walled clear bottom microplates (Costar, Corning, NY, USA) and incubated for 48 h until confluent. The cells were washed three times with PBS (200 μl/wash) and 50 μl of the PBS was retained after the final wash. Quercetin was then added to the cells at different concentrations, and after 10 min of incubation the absorbance of the plate was at 535 nm. For chloride channels-mediated iodide influx assay, the fluorescence of the plate was recorded continuously (200 ms/point) for 2 s (baseline), and 28 s after adding 120 μl PBS, in which the chloride in it was replaced with iodide. Cells treated with PBS only were set as negative control, whereas those treated with 100 μM ATP plus 100 μM CCh were set as positive control.

### Short-circuit current

Snapwell inserts containing HT-29 or ANO1-expressing FRT cells were mounted in an Ussing chamber system (Physiologic Instruments, San Diego, CA). For HT-29 cells, both hemi-chambers contained a standard bathing solution (in mM: 119 NaCl, 1.2 CaCl_2_, 0.6 KH_2_PO_4_, 2.4 K_2_HPO_4_, 1.2 MgCl_2_, 21 NaHCO_3_, 10 D-glucose). For FRT cells, the basolateral chamber was filled with 5 ml of HCO_3_^−^ buffered solution (in mM: 120 NaCl, 5 KCl, 1 MgCl_2_, 1 CaCl_2_, 5 HEPES, 25 NaHCO_3_, 10 D-glucose) and half-Cl^−^ solution was used by replacing NaCl with Na-gluconate in the apical side. The basolateral membrane was permeabilized with 250 μg/ml amphotericin B before the addition of E_act_ and quercetin. All the cells were bathed for 15 min (to ensure a stable baseline before adding the agonist) at 37°C and aerated with 95% O_2_/5% CO_2_ to maintain a pH of 7.4. Short-circuit current was recorded continuously using a VCC MC6 multi-channel voltage-current clamp (World Precision Instruments, Sarasota, FL) with Ag/AgCl electrodes and 3M KCl agar bridges.

ICR mice were anesthetized with sodium pentobarbital and then sacrificed by cervical dislocation. The ileum and colon were removed and washed with ice-cold Krebs bicarbonate solution (in mM: 120 NaCl, 5 KCl, 2.5 CaCl_2_, 1.2 KH_2_PO_4_, 1.2 MgCl_2_, 25 NaHCO_3_, 10 D-glucose). The ileum and colon were opened along the mesenteric border and mounted in Ussing chambers after stripping off the muscularis. Chambers were filled with Krebs bicarbonate solution containing 10 μM indomethacin (to prevent prostaglandin generation) and bubbled with 95% O_2_/5% CO_2_ at 37°C. Amiloride (10 μM) was added to the mucosal side to inhibit the activity of epithelial Na^+^ channel (ENaC).

### Fluid secretion in mouse intestinal closed-loops

ICR mice were starved for 24 h and killed immediately. The small intestines of the animals were then removed, and three closed mid-ileum loops (length 15–20 mm) were generated with sutures. The closed loops were soaked in chambers containing Krebs bicarbonate solution containing quercetin (200 μM) with or without CaCC_inh_-A01 (100 μM). The solution was bubbled with 95% O_2_/5% CO_2_ at 37°C during the whole process. After 4 h, the ileum loops were removed, and the loop length and weight were measured to quantify the net fluid secretion. Fluid secretion was measured separately as loop weight/length ratio.

### Gastrointestinal motility

ICR mice were starved for 24 h and then intraperitoneally administered 100 μl of saline, 200 μM quercetin, 50 μM E_act_ or 200 μM quercetin plus 50 μM E_act_. Fifteen minutes later, the animals were orally administered 10% activated charcoal (diluted in 5% gum arabic) over a 30-min period. The animals were then sacrificed and the small intestines were removed. The peristaltic index was calculated as the percentage of distance that the activated charcoal had traveled relative to the total length of small intestine.

### Statistical analysis

All data were shown by representative traces or expressed as means ± SEs from four to six independent experiments. One-way or two-way ANOVA followed by Dunnett's multiple comparison test was used to compare test and control values, and differences between test and control values were considered statistically significant at the *P* < 0.01 and *P* < 0.05 levels.

### Ethics statement

All animals in this study were handled in accordance with the recommendations of “Guide for the Care and Use of Laboratory Animals of the National Institutes of Health,” and experimental protocol was approved by the Liaoning Normal University Committee on Animal Research. All surgical procedures were performed under sodium pentobarbital anesthesia to minimize suffering.

## Results

### Activation of Cl^−^ transport by quercetin

**Figure 1:**
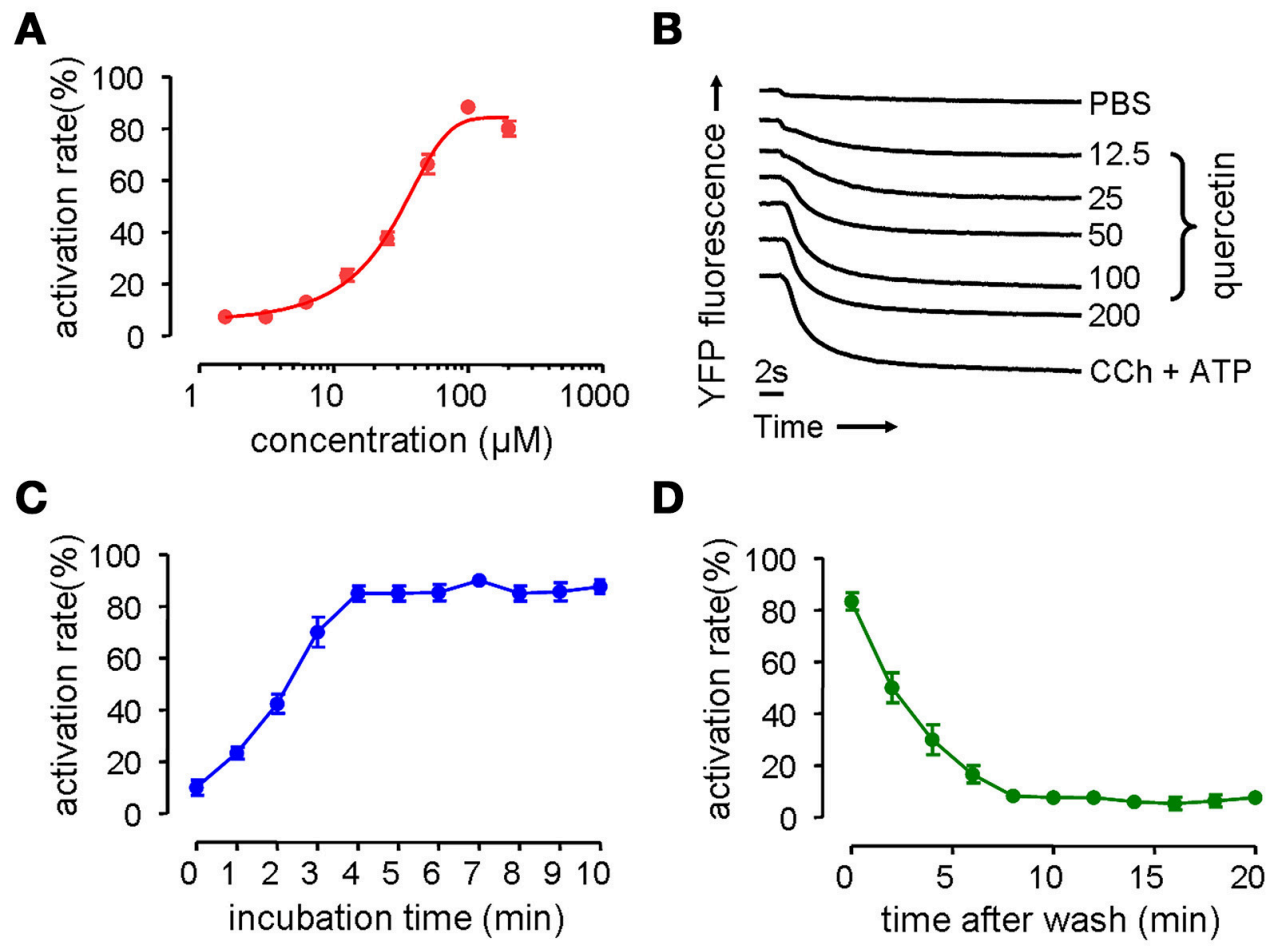
**Activation on chloride channel activity by quercetin. (A)** Dose-dependent activation of chloride channel by quercetin. **(B)** Original traces showing the quenching of YFP fluorescence by I^−^ influx by PBS, quercetin, and CCh plus ATP. **(C)** Time course activation of chloride channel by quercetin. **(D)** Reversal of chloride channel activation following the removal of quercetin (*n* = 5).

Phenotype-based fluorescence quenching test was conducted with HT-29 cells expressing halide sensor fluorescence protein to evaluate the dose-response relationship, kinetics and reversible effect of quercetin on the activation of CaCC. HT-29 cells treated with quercetin exhibited an increase in I^−^ influx, and the increase was dose-dependent, yielding an EC_50_ value of ~37 μM (Figure 1A). Maximal activation was obtained with 200 μM quercetin as seen from the maximum fluorescence quenching in Figure 1B. Carbachol and ATP elevated the intracellular calcium concentration by combining with muscarinic and purinergic receptors, resulting in the activation of chloride channels. The effect exerted by 200 μM quercetin on chloride channel was similar to that produced by a mixture of ATP and carbachol. The activation of chloride channels by 100 μM quercetin was rapid reaching a maximum after 4 min (Figure 1C). Activation of chloride channels by quercetin was reversible, since it was fully abolished 8 min after the removal of quercetin (Figure 1D). These results suggested that quercetin could activate Cl^−^ transport in HT-29 cells.

**Figure 2:**
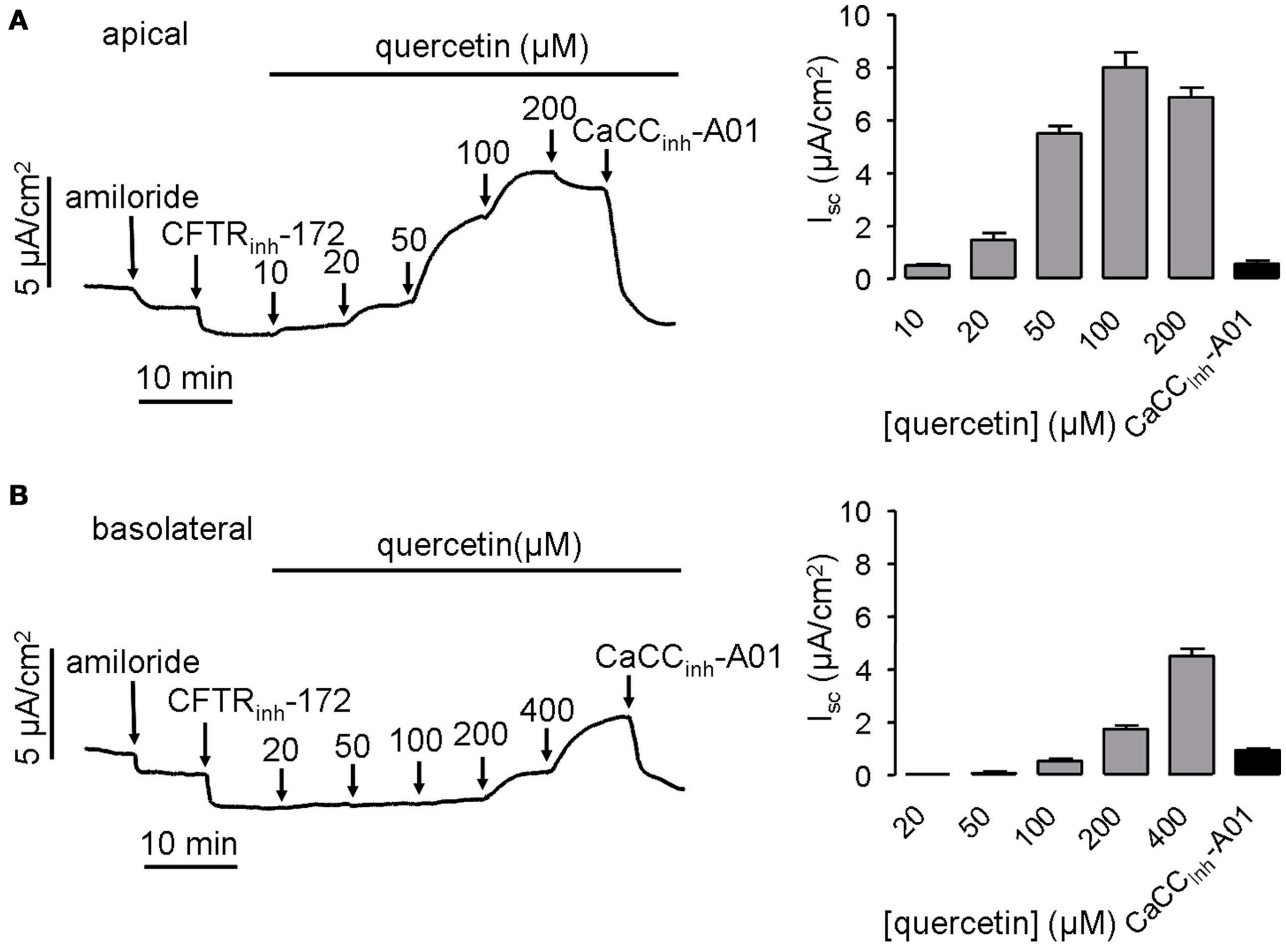
**Activation of CaCC chloride channel activity by quercetin. (A)** Activation of CaCC -mediated Cl^−^ current by apical application of quercetin without or with subsequent addition of 30 μM CaCC_inh_-A01. **(B)** Representative trace of short-circuit current activated by basolateral administration of quercetin without or with subsequent addition of 30 μM CaCC_inh_-A01. The histograms show the magnitudes of short-circuit current obtained from the corresponding traces (*n* = 4).

To confirm the activation of CaCC by quercetin, short-circuit current was further measured in HT-29 cells. Since quercetin also acts as a CFTR chloride channel activator (Pyle et al., 2010; Zhang et al., 2011), 20 μM CFTR_inh_-172 was added to the bath solution before administration of quercetin to eliminate the influence of CFTR-mediated Cl^−^ current. The result showed that quercetin in the apical side of HT-29 monolayers activated the short-circuit currents in a dose-dependent manner. The activation effect could be abolished by the CaCC-specific inhibitor CaCC_inh_-A01 (30 μM) (Figure 2A). In addition, basolateral application of quercetin also activated CaCC-mediated short-circuit current, although this was much less potent than that produced by apical side application (Figure 2B). These results suggested that quercetin can activate both CFTR and CaCC mediated Cl^−^ transport in HT-29 cells.

**Figure 3:**
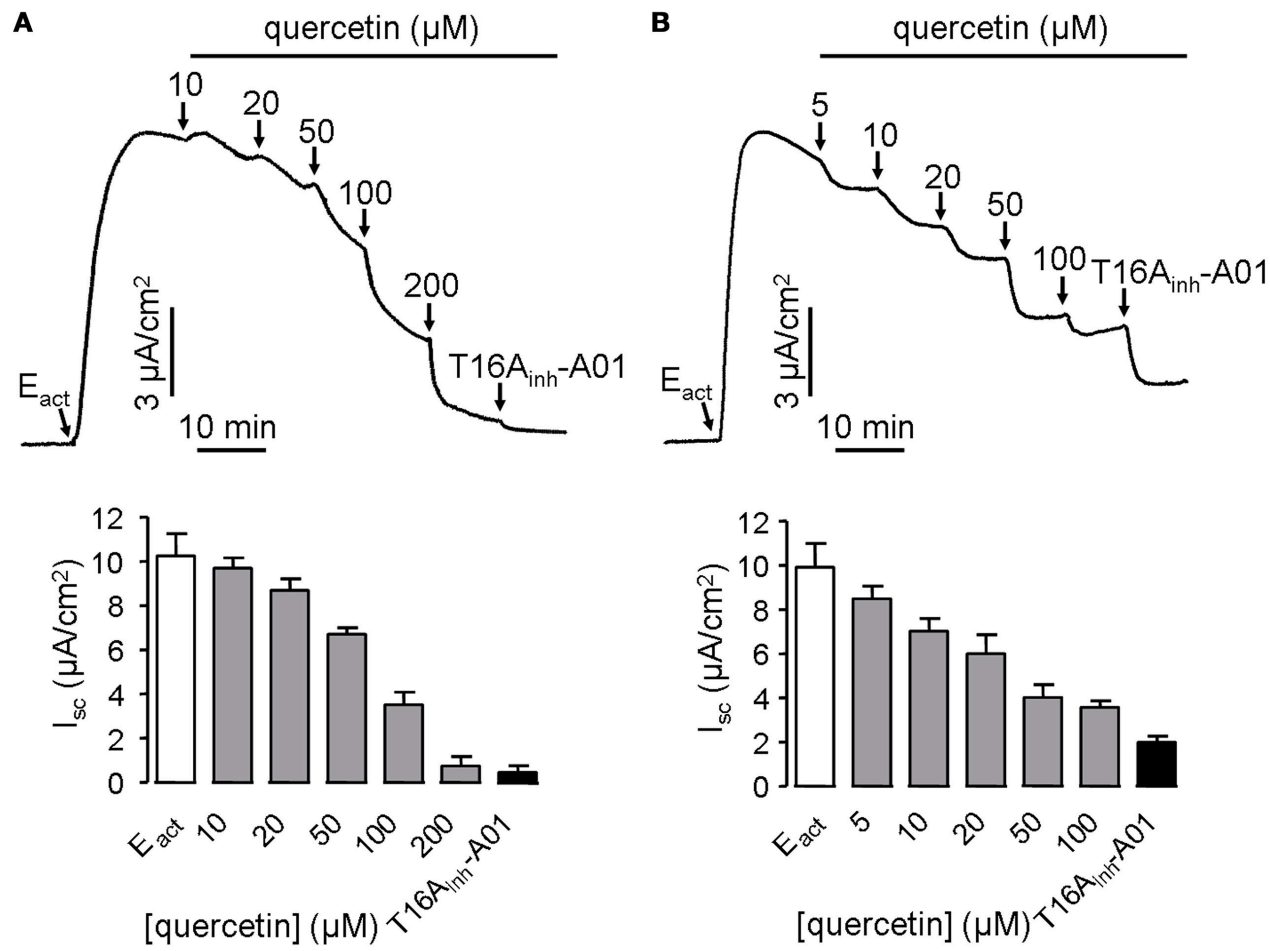
**Inhibitory effect of quercetin on ANO1-mediated short-circuit current in ANO1-expressing FRT cells. (A)** Effect of apical addition of quercetin on E_act_-induced short-circuit current without or with subsequent addition of T16A_inh_-A01. **(B)** Inhibition of E_act_-induced short-circuit current by basolateral application of quercetin without or with subsequent addition of 10 μM T16A_inh_-A01. The histograms compare the magnitudes of inhibition as obtained from the corresponding traces (*n* = 5).

ANO1 is a bona fide calcium-activated chloride channel, thus Cl^−^ current measurement was performed in stably transfected FRT cells expressing ANO1 to investigate the effect of quercetin on ANO1 Cl^−^ channel activity. Surprisingly, quercetin did not activate ANO1-mediated Cl^−^ current in the stably transfected FRT cells. On the contrary, after CaCC current was induced by 10 μM of the ANO1 agonist E_act_, quercetin exhibited inhibitory activity. When added to the apical side of FRT monolayers, quercetin slightly inhibited the current at low concentrations (10–20 μM) and almost completely inhibited the Cl^−^ current at 200 μM (Figure 3A). The remaining current could be inhibited by the specific inhibitor T16A_inh_-A01. Basolateral administration of quercetin resulted in partial inhibition of E_act_-induced short-circuit current (~57%) (Figure 3B). The inverse effects of quercetin on ANO1 in transfected FRT cells and CaCC current in HT29 cells suggested the existence of calcium-activated chloride channels in the intestinal epithelial cells that are different from ANO1.

**Figure 4:**
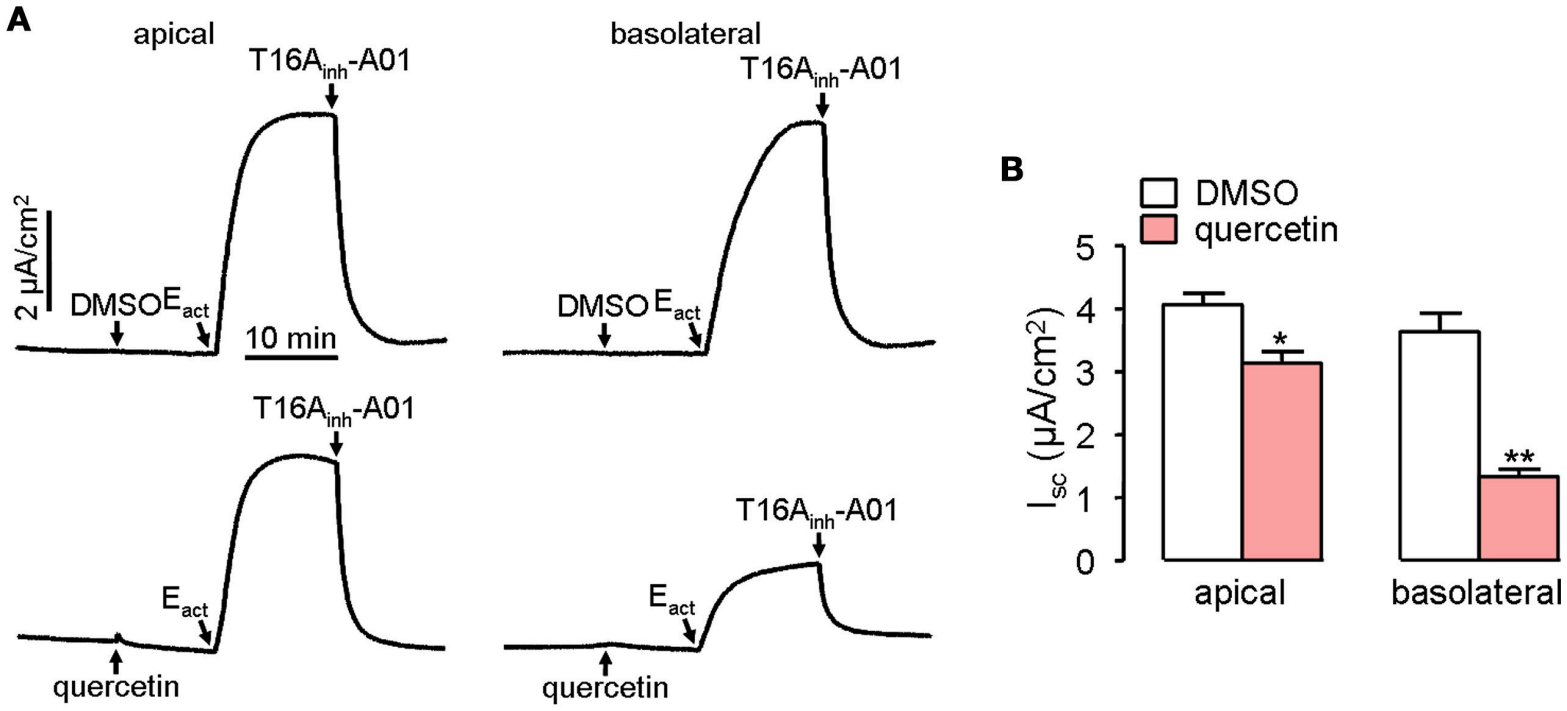
**Inhibitory effect of quercetin on ANO1-mediated Cl^**−**^ transport. (A)** Short-circuit current induced by E_act_ following DMSO addition in apical or basolateral side (upper panel). Partial inhibition of ANO1-mediated short-circuit current by apical or basolateral pre-treatment of 100 μM quercetin (lower panel). **(B)** Magnitudes of short-circuit current inhibition generated by apical vs. basolateral application of quercetin. Data are the means ± SEs from four determinations. “^*^” and “^**^” indicate significantly difference from control at the *P* < 0.05 and *P* < 0.01 levels.

Short-circuit current experiment was further performed to investigate whether quercetin act as a blocker of ANO1 chloride channel activity in ANO1-expressed FRT cells. Pre-treatment of both the apical and basolateral sides of FRT cells with 100 μM quercetin partially blocked the Cl^−^ current induced by E_act_, yielding an inhibition rate of ~20 and 65%, respectively for apical and basolateral sides compared to the control (Figure 4).

### Characterization of CaCC activation by quercetin

**Figure 5:**
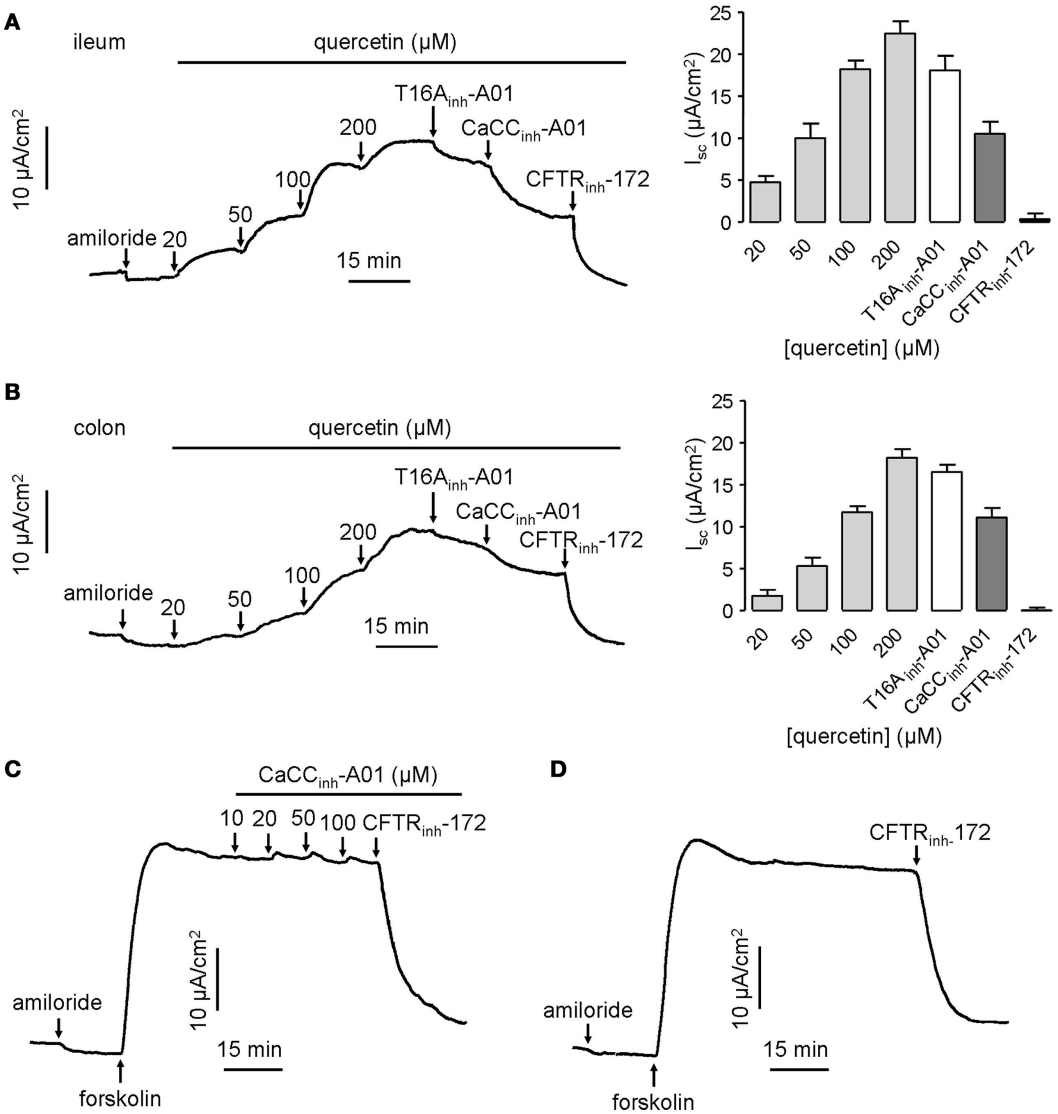
**Activation effect of quercetin on chloride channel activities in mouse ileum and colon. (A)** Activation of CFTR and CaCC-mediated Cl^−^ current in freshly isolated mouse ileum by different concentrations of quercetin. **(B)** Activation of CFTR and CaCC-mediated Cl^−^ current in colonic epithelia by different concentrations of quercetin. The corresponding histogram of each trace compares the *I*_*sc*_ obtained from different concentrations of quercetin. **(C)** Inhibition of CFTR-mediated Cl^−^ current by indicated CaCC_inh_-A01 in mouse ileum. **(D)** Inhibition of CFTR chloride channel activity by CFTR_inh_-172 (100 μM).

CaCCs have been shown to exist in mouse intestinal epithelia, and therefore the efficacy of quercetin was tested *ex vivo* in isolated mouse intestinal mucosa by Ussing chamber short-circuit assay. The results showed that the short-circuit currents in mouse ileal and colonic epithelia on serosal side were increased by quercetin in a dose-dependent manner, reaching a peak at 200 μM in both cases (Figures 5A,B). *I*_*sc*_ stimulated by the same concentration of quercetin was slightly higher in mouse ileal mucosa than in colonic mucosa. As expected, the current was slightly inhibited by 20 μM T16A_inh_-A01 (inhibition rate ≤ 20%), but was partially inhibited by 100 μM CaCC_inh_-A01 (inhibition rate ≥ 40%) while the remaining current was abolished by 100 μM CFTR_inh_-172. The inhibitory effect of CaCC_inh_-A01 on CFTR chloride channel activity in mouse ileal mucosa was also measured, and CaCC_inh_-A01 did not appear to inhibit forskolin-induced CFTR-mediated Cl^−^ current at 10–100 μM (Figures 5C,D). These results further confirmed that quercetin activated a CaCC current different from ANO1 in mouse intestinal epithelia.

**Figure 6:**
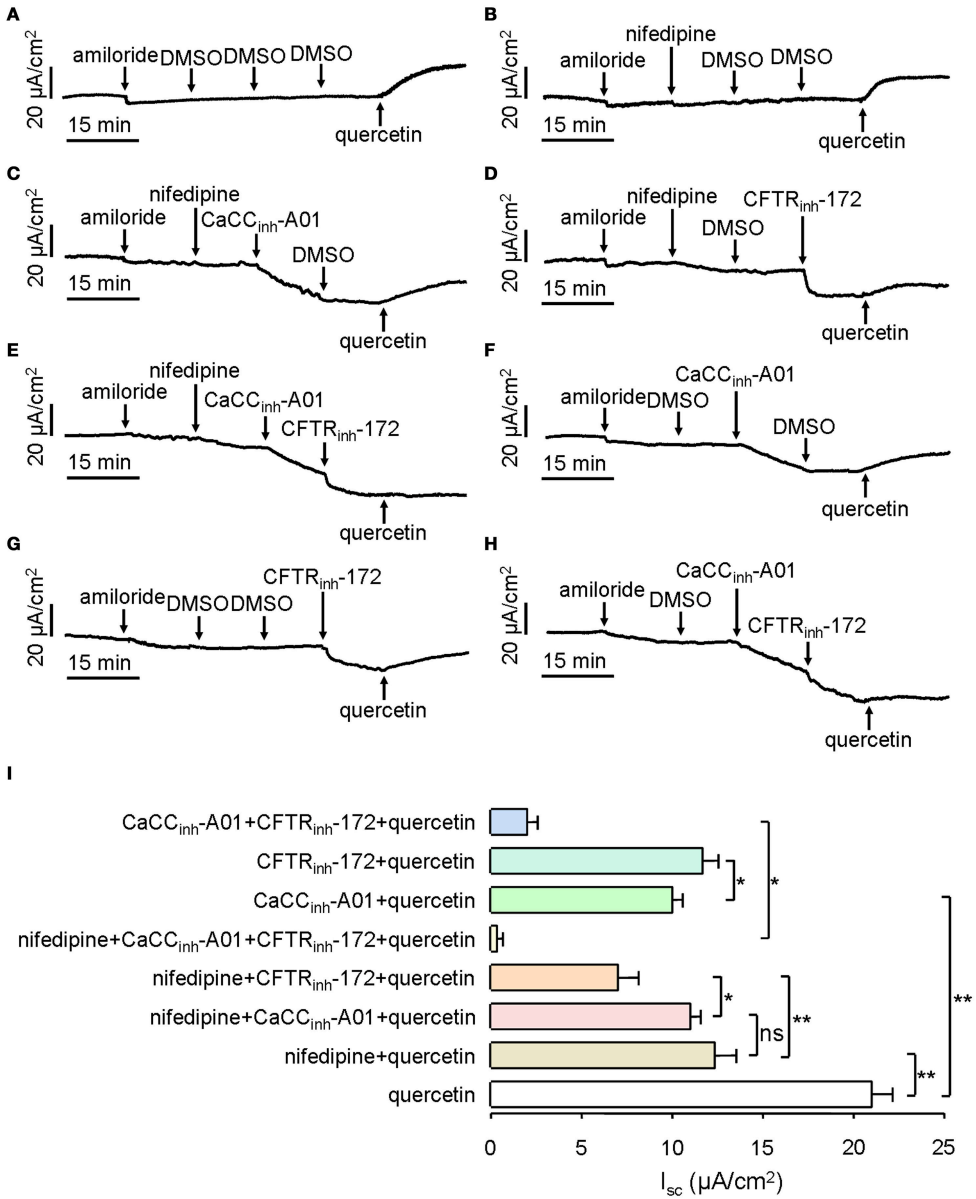
**Characterization of quercetin-induced short-circuit current in mouse ileum and colon. (A–H)** Activating effect of quercetin after pre-treatment with DMSO **(A)**, nifedipine **(B)**, nifedipine plus CaCC_inh_-A01 **(C)**, nifedipine plus CFTR_inh_-172 **(D)**, nifedipine plus CaCC_inh_-A01 plus CFTR_inh_-172 **(E)**, CaCC_inh_-A01 **(F)**, CFTR_inh_-172 **(G)**, and CFTR_inh_-172 plus CaCC_inh_-A01 **(H)**. **(I)** Comparison of the inhibitory effects of different agents on the short-circuit currents. Data are the means ± SEs from 6 to 7 determinations. “^*^” and “^**^” indicate significantly difference from control at the *P* < 0.05 and *P* < 0.01 levels.

Next, we investigated the components of quercetin-induced short-circuit current in mouse ileum. CaCC can be activated by intracellular Ca^2+^, and Ca^2+^ influx by calcium channels is a main route to enhance intracellular calcium concentration. In order to prove whether L-type calcium channels were involved in quercetin-induced CaCC activation, 10 μM of an L-type calcium channel inhibitor (nifedipine) was added to the serosal incubation solution. Compared with DMSO control (Figure 6A), the short-circuit current induced by 200 μM quercetin was reduced by 41% after pre-treatment with nifedipine (Figure 6B). The result suggested that L-type calcium channel was involved in the *I*_*sc*_ generated by quercetin. We also analyzed the effect of CaCC_inh_-A01 and CFTR_inh_-172 on quercetin-induced *I*_*sc*_ after inhibition of the L-type calcium channel. Quercetin-stimulated short-circuit current was further decreased by ~6.3 and 43% after the addition of nifedipine plus CaCC_inh_-A01 (100 μM) and nifedipine plus CFTR_inh_-172 (100 μM), respectively (Figures 6C,D). No significant difference was observed between the nifedipine group and nifedipine plus CaCC_inh_-A01 group (Figures 6B,C). These results indicated that Ca^2+^ influx through L-type calcium channel was an important way to activate intestinal epithelial CaCC. As CFTR_inh_-172 could further inhibit the short-circuit current by 25% (Figure 6D), it suggested the existence of CFTR-mediated Cl^−^ current. However, CaCC_inh_-A01 or CFTR_inh_-172 could only inhibit part of the *I*_*sc*_ induced by quercetin. CaCC_inh_-A01 produced a slightly higher inhibition rate (~52%) (Figure 6F) than CFTR_inh_-172 (~44%) (Figure 6G). These results were consistent with previous findings. In addition, pre-treatment with nifedipine, CaCC_inh_-A01 and CFTR_inh_-172 almost completely abolished quercetin-induced short-circuit current in mouse ileal tissues (Figure 6E), but there remained a small amount of short-circuit current after the addition of CaCC_inh_-A01 and CFTR_inh_-172 (Figure 6H). Figure 6I summarizes the result of *I*_*sc*_ from the different treatments. The results suggested that quercetin-induced short-circuit currents may involve other Ca^2+^-activated channels such as Ca^2+^-activated K^+^ channel.

**Figure 7:**
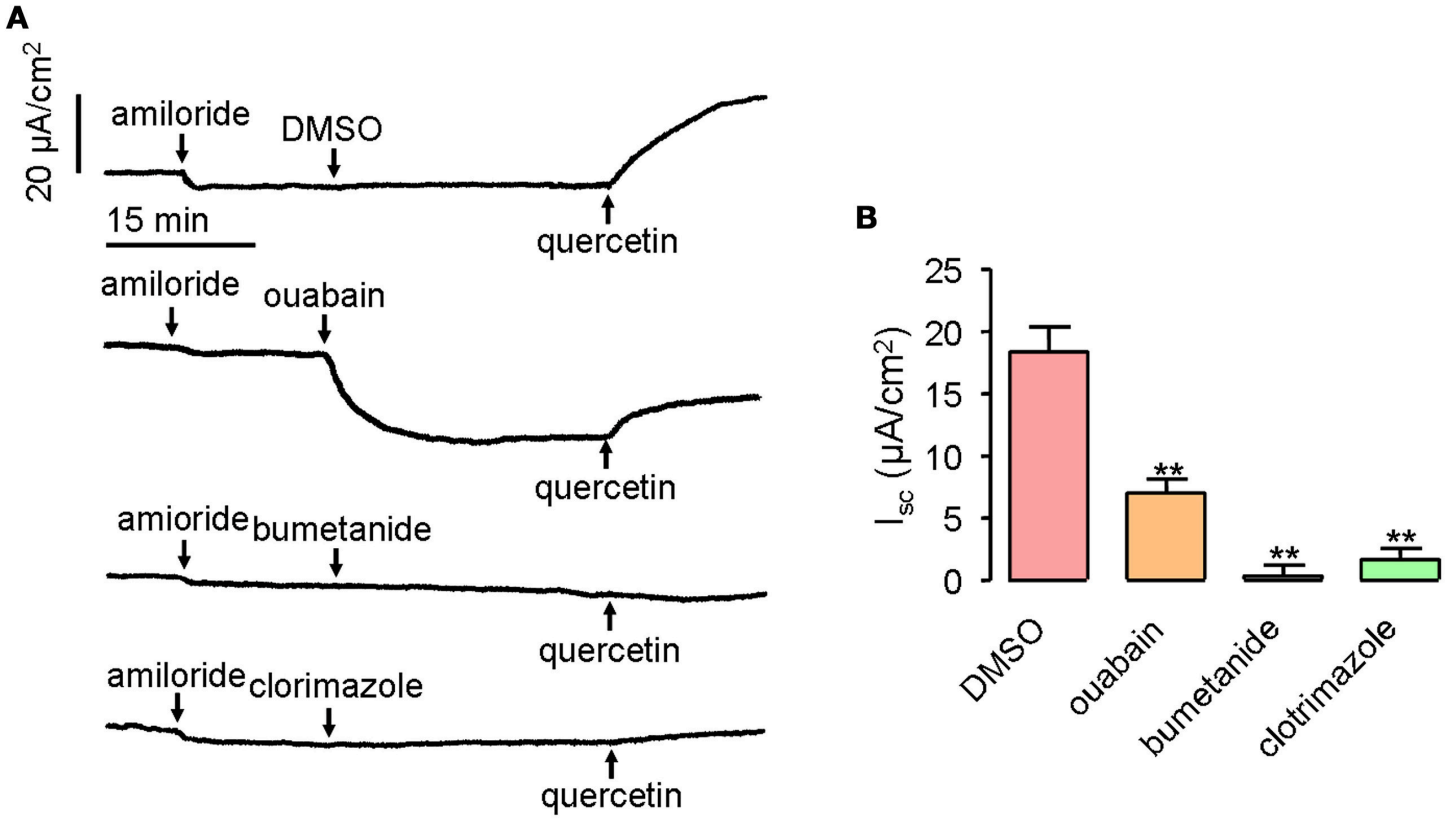
**Inhibition of quercetin-induced short-circuit currents by basolateral ion channel and transporter inhibitors. (A)** Traces showing the inhibition of quercetin-induced short-circuit currents by ouabain, bumetanide and clotrimazole. **(B)** Comparison of the inhibitory effect of different inhibitors on quercetin-induced short-circuit currents. Data are the means ± SEs from four experiments. “^**^” indicate significantly difference from control at the *P* < 0.01 levels.

Transepithelial Cl^−^ secretion can be promoted by activated chloride channel, as well as the accumulation of Cl^−^ in the epithelial cells. Therefore, it was important to determine whether transporters and ion channels in the basolateral membrane would participate in quercetin-activated short-circuit currents. Ouabain applied at 1 mM can inhibit 61% of the quercetin (200 μM)-activated short-circuit current when applied to mouse ileum on the serosal side, whereas bumetanide and clotrimazole given at 100 and 50 μM, respectively, completely abolished the short-circuit currents stimulated by quercetin (Figures 7A,B). These results suggested that Na^+^/K^+^-ATPase, NKCC, and K^+^ channels could participate in quercetin-induced Cl^−^ currents.

### Activation of intestinal fluid secretion in mouse ileum by quercetin

**Figure 8:**
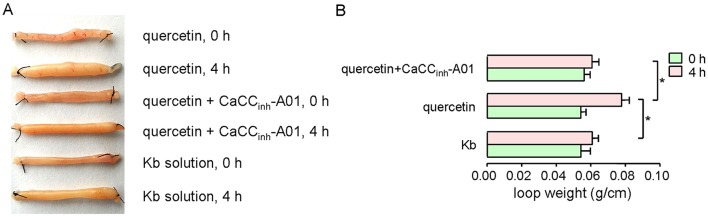
**Activation of ileal fluid secretion by quercetin. (A)** Images of ileal loops co-incubated with quercetin, quercetin plus CaCCinh-A01, and Krebs bicarbonate solution at 0 and 4 h. **(B)** Histogram comparing the loop weight/length ratios from different treatments. Data are the means ± SEs from four experiments. “^*^” indicates significant difference from the control at the *P* < 0.05 level.

The effect of quercetin on intestinal fluid secretion was investigated by closed-loop experiments. Figure 8 shows the ileal closed-loops incubated with 200 μM quercetin, and 200 μM quercetin plus 100 μM CaCC_inh_-A01. Krebs bicarbonate solution was used as a control in this test. Quercetin significantly increased fluid secretion in the closed loop, which was reduced by CaCC_inh_-A01. The results revealed that quercetin induced intestinal fluid secretion via activation of CaCC chloride channel activity.

### Inhibition of gastrointestinal motility by quercetin in mouse

**Figure 9:**
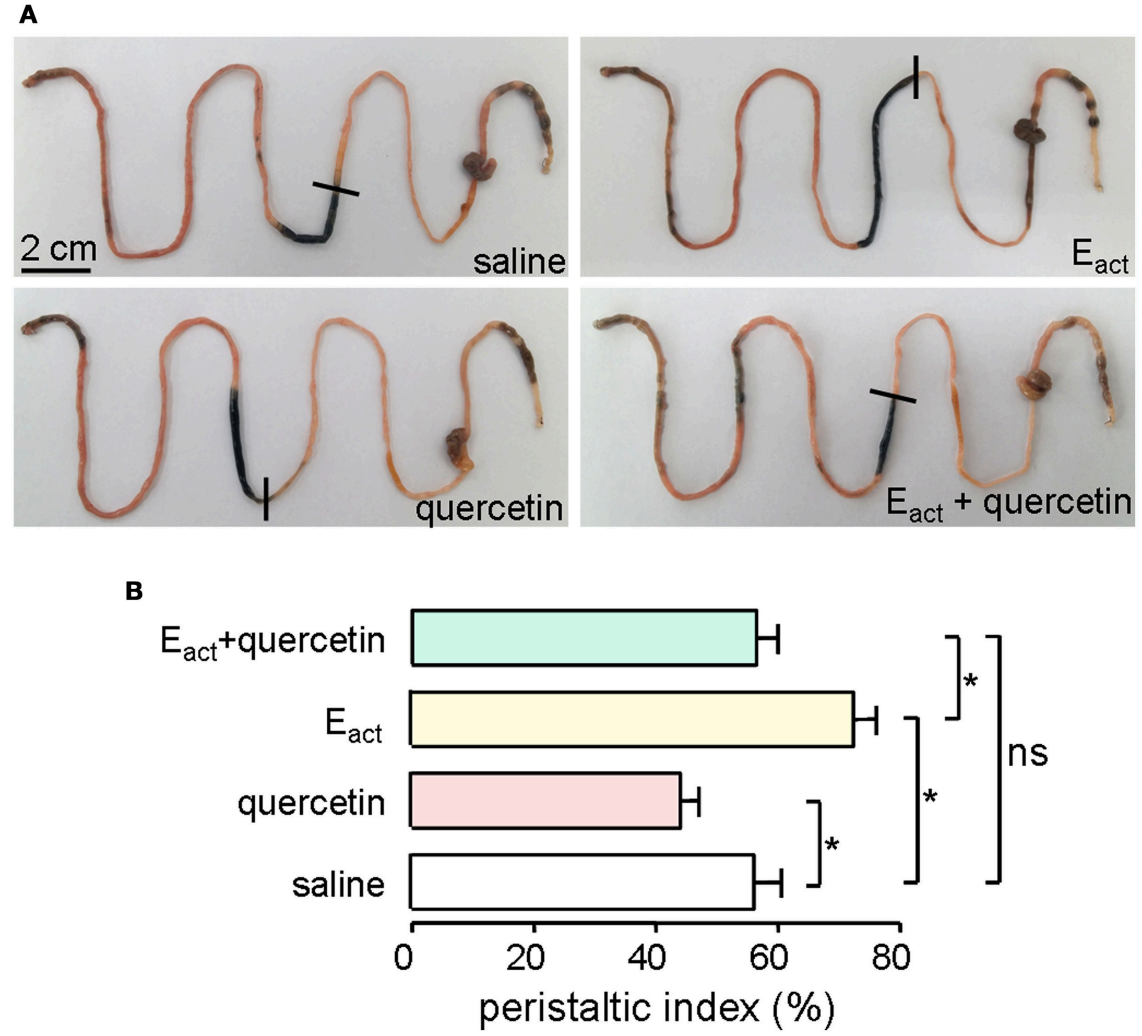
**Inhibition of gastrointestinal movement by quercetin in mice. (A)** Photographs of isolated mouse intestines showing traveled distance of activated charcoal after intraperitoneal administration of saline (negative control), E_act_ (50 μM), quercetin (200 μM) or quercetin (200 μM) plus E_act_ (50 μM). **(B)** Histogram comparing the peristaltic indexes from different treatments. Data are the means ± SEs from five experiments, ^*^indicates the significant difference from control at the *P* < 0.05 level.

ANO1 is expressed in the interstitial cells of Cajal in the intestine where it modulates smooth muscle contraction (Huang et al., 2009; Hwang et al., 2009; Ferrera et al., 2010). Thus, intestinal motility measurement was performed in mice to evaluate the efficacy of quercetin *in vivo*. Intraperitoneal administration of 50 μM of the ANO1 activator E_act_ accelerated intestinal peristalsis, with peristaltic index of 72 ± 5.5% compared to saline control of 56 ± 6.9%. As shown in Figure 9A, quercetin (200 μM) significantly inhibited the basal and E_act_-stimulated intestinal peristalsis. Figure 9B compares the peristaltic indexes from the different treatments. These results demonstrated that quercetin slowed intestinal peristalsis by inhibiting ANO1 activity.

## Discussion

Flavones are multifunctional ubiquitous compounds extracted from various plants. Recent studies have established that flavones have many biological activities, such as anti-inflammatory, antiviral, anti-atherosclerotic and anticancer activities (Manthey et al., 2001). Flavones also have an effect on cell cycle, apoptosis, ion transport, and gene expression (Birt et al., 2001; Hsu et al., 2001). As flavones have a wide range of activities and are present in natural abundance, they have become an attractive dietary supplement. It has been well documented that flavones (including quercetin) can activate CFTR chloride channel (Pyle et al., 2010; Zhang et al., 2011). The major finding of the present study was the identification of CaCC chloride channels as new molecular targets of quercetin.

The identification of quercetin as intestinal CaCC modulator would highlight the possibility of different types of CaCC proteins in the intestine. In previous studies, Verkman's group has developed a phenotype-based screening assay to screen for CaCC chloride channel inhibitors using HT-29 cells (De La Fuente et al., 2008). Although the exact molecular identities of CaCC_GI_ is still unclear, a much lower expression level of ANO1 in HT-29 cells has been observed (Wanitchakool et al., 2016). In the present study, we found that quercetin could activate CaCC in both HT-29 cells and mouse ileum and colon. However, quercetin has inhibitory effect on ANO1 chloride channel activity in ANO1-expressing FRT cells, suggesting the existence of different types of CaCC in intestinal epithelial cells.

Ileal and colonic epithelia express CaCC_GI_ and ANO1 (Schreiber et al., 2010), so the stimulatory effect of quercetin on intestinal epithelial Cl^−^ secretion was also examined. The results (Figures 5A,B) were in accordance with the short-circuit current results obtained from HT-29 cells. This not only proved the existence of CaCC_GI_, but also suggested that the pharmacological activities of quercetin in intestinal epithelia included the promotion of calcium-activated chloride secretion mediated by CaCC_GI_ rather than ANO1.

Fluid secretion across intestinal epithelia contributes to digestion, stool passage, prevention of microbial infection and regulation of body fluid content. A lack of fluid secretion may therefore lead to constipation. The process of fluid secretion is driven by epithelial Cl^−^ secretion through chloride channels in the enterocytes (Barrett and Keely, 2000; Kiela and Ghishan, 2009). Chloride secretion across the intestinal epithelia occurs mainly through Cl^−^ channels including CFTR, CaCC and ClC-2, among which CFTR and CaCC may play pivotal roles (Murek et al., 2010). It has been reported that CaCC_GI_ is responsible for the intestinal fluid accumulation in rotaviral enterotoxin-stimulated diarrhea (Ko et al., 2014). Our data showed that the weight/length ratio of ileal closed-loops treated with quercetin increased by 41%, while CaCC_inh_-A01 decreased the ratio to 16% (Figure 8B). It has been reported that quercetin could activate CFTR Cl^−^ channels in enterocytes (Pyle et al., 2010; Zhang et al., 2011) and this may consequently lead to fluid accumulation in the intestine, thus activation of CFTR and CaCC by quercetin may contribute to the treatment of constipation.

The main function of interstitial Cajal cell (ICC) is to participate in gastrointestinal slow wave generation and transmission, linking sensory nerves to smooth muscle cells. As ANO1 is the iconic protein of ICC, inhibition of ANO1 may delay the intestinal movement and thus increase the time for fluid absorption (Hwang et al., 2009). Hwang and colleagues reported that pharmacological inhibition or genetic deletion of ANO1 abolishes slow waves in murine small intestine (Hwang et al., 2009). Likewise, we showed that quercetin could significantly reduce gastrointestinal motility in mice, suggesting that inhibition of ANO1 chloride channel activity may partly account for the inhibitory effect of quercetin in intestinal contraction. The ability of quercetin to delay intestinal peristalsis could be of great use to the treatment of intestinal dynamic disorder.

CaCC activation can be acquired by improving the cytoplasmic Ca^2+^ concentration. Quercetin is known to stimulate insulin secretion by increasing Ca^2+^ influx through stimulation of L-type Ca^2+^ channels (Bardy et al., 2013). Cav1.3 is a voltage-dependent L-type Ca^2+^ channel, which is expressed in the intestine and plays a pre-dominant role in the intake of Ca^2+^ during intestinal ingestion (Thongon et al., 2009). As pre-treatment of mouse ileum with the Ca^2+^ channel inhibitor nifedipine reduced quercetin-induced Cl^−^ current (Figure 6I), quercetin may activate CaCC chloride channel activity through stimulation of L-type calcium channel-mediated Ca^2+^ influx.

The transport of chlorides requires the electrochemical driving force established by Na^+^/K^+^-ATPase, NKCC, and K^+^ channels in the basolateral membrane. Some investigators believe that chloride secretion by quercetin is due to the activation of CFTR and basolateral NKCC (Niisato et al., 2004). Besides, at least part of the quercetin-induced Cl^−^ secretion can be explained by activation of basolateral K^+^ channels (Cermak et al., 2002). This may account for the activation of Cl^−^ secretion by serosal application of quercetin in intestinal epithelia. Quercetin-stimulated Cl^−^ secretion was partly decreased by the Na^+^/K^+^-ATPase inhibitor ouabain (Figure 7), but completely abolished by the NKCC inhibitor bumetanide and K^+^ channel inhibitor clotrimazole. These results suggested the important role of Na^+^/K^+^-ATPase and K^+^ channels in quercetin-induced Cl^−^ secretion.

Constipation is a common clinical symptom, which caused serious impact on the patient's quality of life. CFTR and CaCCs chloride ion channels have become important targets for the treatment of constipation in recent years. Experimental results showed that mechanisms of lubiprostone and linaclotide approved in the treatment of chronic constipation in adults and irritable bowel syndrome were due to their activation effect of chloride channels (Busby et al., 2010; Fei et al., 2010; Castro et al., 2013). Therefore, quercetin simultaneously activated intestinal epithelial CaCCs and CFTR chloride channels could be used in the treatment of constipation. However, because quercetin has no effect on the mucosal side in mouse intestinal short-circuit current assay, it may not have therapeutic efficacy by oral application.

## Conclusion

The present study revealed for the first time that quercetin, a naturally occurring flavone, could modulate the activity of intestinal CaCC chloride channels. The inverse effects of quercetin on ANO1 and CaCC_GI_ indicated the existence of non-ANO1 type CaCC chloride channels in intestinal epithelia. It also uncovered a new pharmacological target of flavonoids, highlighting the potential therapeutic strategy for treating CaCC-related diseases like constipation, secretory diarrhea and hypertension.

## Author contributions

BY designed the project, performed experiments, analyzed and discussed the data, wrote the manuscript. YJ performed some experiments, analyzed data and wrote the manuscript. LJ performed some experiments. TM and HY designed the project, analyzed and discussed the data, wrote the manuscript.

### Conflict of interest statement

The authors declare that the research was conducted in the absence of any commercial or financial relationships that could be construed as a potential conflict of interest.
